# Building a diverse national research advisory board

**DOI:** 10.1017/cts.2024.1169

**Published:** 2025-01-09

**Authors:** Gayle Valensky, Jeff B. Pawelek, Lauren Serpico, David Rodriguez, Maribel Pieters, Yulissa Perez, Julia Moore Vogel

**Affiliations:** Scripps Research Translational Institute, Scripps Research, La Jolla, CA, USA

**Keywords:** Community-based participatory research, community outreach, underrepresented in biomedical research, community advisory board, virtual technologies, diversity and inclusion in clinical trials

## Abstract

In healthcare and medical research, advisory boards are now commonplace, but most boards consist of a relatively homogenous, geographically collocated group, often demonstrating demographic imbalance. It is crucial to include individuals from diverse backgrounds on community advisory boards for healthcare and medical research to address ongoing health disparities and ensure studies are more culturally competent so that we can achieve more inclusive representation. We conducted purposeful recruitment to attract a demographically diverse group of community members across the United States (U.S.) to partner with the *All of Us* Research Program to inform our strategies including program recruitment, engagement, retention, and incentives. Recruitment of a diverse group of advisors and purposeful community building has created a psychologically safe environment where members openly share their opinions, thoughts, and perspectives to shape various aspects of this ambitious, nationwide research program.

## Introduction

There is widespread recognition of the persistent health disparities and inequities in our society, and it is well-documented that these disparities negatively impact racially and ethnically marginalized populations. The *All of Us* Research Program aims to help address this by enrolling 1 million or more participants from diverse backgrounds to gather data on genetic, environmental, social, and behavioral factors to better understand how these factors impact health outcomes [1]. These data will be broadly shared with qualified researchers to accelerate medical breakthroughs. Without meaningful involvement from diverse communities of which *All of Us* is recruiting, the project would face significant challenges to sustain 80% representation of groups historically underrepresented in biomedical research (UBR).

Individuals from UBR groups have long experienced substandard health outcomes as a result of health care and research enterprises inadequately serving diverse patient populations [2,3]. One of the primary strategies of forging new relationships, establishing trust, bestowing respect, and increasing participation in biomedical research in diverse communities is through community-based participatory research (CBPR) [4]. This framework places community at the center of the research planning and execution process and includes a formal mechanism to communicate with key community members to get their input, feedback, and opinions about how the research will be carried out [5,6]. Benefits from the CBPR model include increased trust between communities and academic institutions, reduction of communication and collaboration barriers, incorporation of novel research methodologies, community empowerment, increased diversity, and greater likelihood of participant retention [7].

One of the motivations behind CBPR stems from overall low participation across all demographics in clinical research [8]. For example, less than 10% of cancer patients participate in clinical trials [9] and nearly 1 in 5 clinical trials fail to achieve accrual goals [10]. Additionally, there is a persistent lack of diversity in clinical research due to a variety of barriers that have been well-described in the literature [11,12]. There are valid reasons why people choose not to participate, including lack of awareness of study opportunities, burden of participation, financial barriers, and fear of study-related risks [13,14]. To overcome these challenges, CBPR connects community members with researchers to integrate community-identified needs and perspectives into a more inclusive, participant-centric study design. Since its introduction, CBPR has been adopted by the National Institutes of Health, professional medical societies, and academic medical centers to promote community involvement and population diversity in clinical research [15–17].

The formation of a community advisory board (CAB) is a proven CBPR-based approach investigators use to engage key members of the community to take part in the research design process [18–22]. CABs are not always designed to include individuals who come from different socioeconomic, racial/ethnic, educational, and cultural backgrounds which limits their utility to help investigators develop a more nuanced perspective of their target study population, including perceptions and barriers for participation in research. Since CABs often lack diversity [23–26], they can result in limited perspectives, bias in decision-making, perpetuation of health disparities, and reduced trust and engagement in medical institutions. Similar to the makeup of corporate boardrooms, increasing diversity on CABs can improve psychological safety and quality of feedback by reducing feelings of tokenism and increasing feelings of inclusion [27,28]. This can be measured by including multiple members of diverse communities and asking CAB members to complete a feedback form after each meeting to assess their satisfaction with meetings and their contributions.

A core tenet of *All of Us* is to treat participants as partners, therefore, each program awardee is required to establish a CAB with members representative of the diverse *All of Us* cohort[1]. As an *All of Us* awardee, the Scripps Research Translational Institute (SRTI) formed a representative CAB that is formally called the Virtual Advisory Team (VAT). With the engagement and guidance of the VAT, the SRTI team has recruited 20% of the total *All of Us* cohort – roughly 175,000 participants to date, 78% of whom have been historically underrepresented in biomedical research.

In this paper, we describe our rationale and approach to forming the *All of Us* VAT, how we achieved diversity, and how we continue to engage underrepresented communities to join one of the largest, most diverse health research programs in the US.

### Community engagement in health research

Recognizing and respecting the diverse cultural backgrounds of community members is vital for fostering effective collaboration [29]. The process of involving diverse members of the community in identifying common problems, mobilizing resources, and developing and implementing strategies for reaching collective goals has been adapted by social change professionals for decades [30].

Community engagement began to be emphasized in health research in the 1980s during the height of the AIDS epidemic [31] which aimed to broaden access to clinical trials for HIV-infected individuals in underrepresented communities based on being part of a minority group, those who lived far from an established clinical trial site, those who were sexual partners of HIV-infected individuals or IV drug users. Awardees were required to demonstrate community support for this effort. However, even with these requirements, many communities including women, transgender individuals, and those living in Sub-Saharan African nations have been excluded from a majority of HIV research [32]. Without full representation of those from diverse backgrounds, research results will not be generalizable to all who are infected with HIV.

### Overview of the VAT

The overarching purpose of the VAT was to bring diverse community voices into the research design and implementation processes for work being done at SRTI to promote the *All of Us* Research Program. Therefore, VAT members were selected to represent the diversity of the *All of Us* participant community of which 80% must include individuals underrepresented in biomedical research based on race and ethnicity, biological sex at birth, gender identity and sexual orientation, living with physical or mental disabilities, barriers to accessing care, and others from disadvantaged backgrounds including those of low economic status or educational attainment and those from geographically isolated environments. *All of Us* also has the goal for the dataset to include 50% of individuals who are UBR by race/ethnicity [1]. 21 of 28 (75%) of the *All of Us* VAT includes members from underrepresented backgrounds based on race/ethnicity. VAT members were recruited from a network of *All of Us* partners, including patient advocacy groups and community organizations, to create long-term partnerships between researchers and participants.

Through a series of recurring meetings, VAT members are invited to provide structured and unstructured feedback on all aspects of the *All of Us* Research Program including messaging, user experience, self-guided biosample collection, electronic health record sharing, and privacy protection including data security practices.

### Recruitment and membership

The public learned about VAT membership opportunities through partner outreach networks, including through a variety of non-profit organizations, PatientsLikeMe (an online health-focused social platform), and *All of Us* community partner groups [33,34]. Through these networks, the VAT recruitment announcement was shared via email and online forums and remained active for approximately two months. While we risked excluding some members of the community, because of national reach and limited opportunity for meeting in-person on a regular basis, we required VAT members to have internet access, which candidates indicated on the brief, online application form. Upon submission, applications were reviewed and scored by the SRTI team based on their participation in *All of Us* (preferred but not required), and their experience in community advocacy work, health equity, and related topics, and ability and desire to represent one or more UBR groups. Similar to the initial recruitment process, we accept new VAT member applications at the beginning of each calendar year and announce the opportunity through our partner and social networks. Current VAT members are welcome to invite individuals in their networks to apply. Candidates with the highest application scores were invited for a virtual interview with the SRTI team. Applicants were scored on a scale of 1-10 with consideration for previous experience on community advisory boards, identifying as a member of an underrepresented community, having a chronic condition, involvement with community-based organizations, and ability to commit to VAT member responsibilities such as meeting attendance. After virtual interviews, the SRTI team re-reviewed and updated their application score based on their interview performance. Candidates with the highest overall score were offered an opportunity to join the VAT. VAT members receive an annual honorarium and travel funds if they are invited to attend meetings.

### Formation and meeting structure

In May 2018, the VAT was formed with eight members from the community and four staff members from SRTI. All new members receive a welcome packet that lays out the following ground rules for participation:

1. Respect: Treat each other and others’ opinions, ideas, and contributions with respect. Be mindful of language and try not to interrupt each other.
2. Confidentiality: We often share internal documents that can’t be shared or reposted anywhere. What we share outside of social media posts to our page is considered private unless otherwise noted.
3. Honesty: Please share your honest opinion – it will help make us better! With respect and privacy, it will help us to be more open and honest during our meetings.

All meetings are managed by the SRTI staff through internal weekly planning meetings. The VAT members are invited to attend a virtual meeting for one hour and member attendance and participation are voluntary. With the initial VAT membership, meetings were scheduled quarterly, but meeting frequency was changed to every six weeks as the VAT grew to ensure newer members had ample opportunities to pose questions, provide input, and connect socially with the team. We also frequently communicate with members and ask for feedback between meetings. VAT participation is expected for at least one year; however, there is no limit for the number of years an individual can remain on the VAT.

In an effort to feel connected as a community, meetings begin with a wellness check to provide the attendees the opportunity to share how they are feeling or something of interest in their personal lives, such as a holiday, trip, or activity. The wellness check is intended to promote group engagement, allow the members to get to know each other on an individual level, and provide the opportunity for the group to learn from each other. These types of interpersonal exercises helped our team develop a bond, which, as research suggests, positively impacts members’ perception of their participation as well as the longevity of their participation [35]. The wellness check is followed by the main topic of discussion. Meeting topics are generated by four SRTI staff based on the latest research activities being planned, and the broader SRTI team is welcome to generate pertinent discussion topics as well. Topics are generally geared towards seeking feedback from the VAT and can range from deciding on whether to add a new participant survey to how personalized genetic results are disseminated. VAT members can also propose topics which can be included as future agenda topics if deemed valuable for group discussion. For meetings with high attendance, virtual breakout rooms can be used to promote more active participation among attendees and help SRTI staff more effectively moderate the discussions.

All meeting minutes are shared with the VAT membership and subsequently shared with all SRTI *All of Us* staff. VAT members also receive a recap of key points after each meeting, which includes notes, a group photo, and a weblink to access the audio recording.

### Community representation

**Figure 1.**
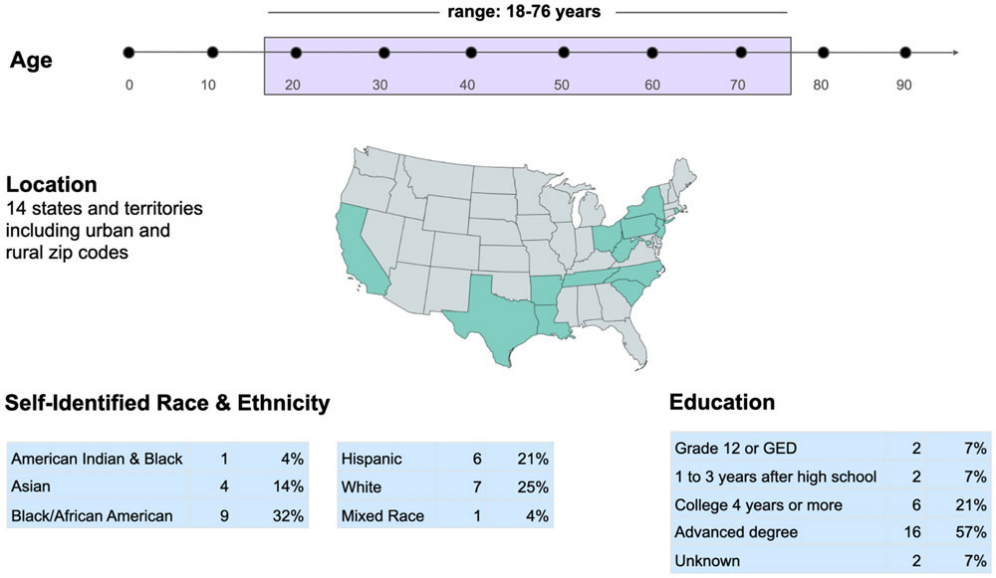
The demographic profile of the 28-member virtual advisory team (VAT) includes 75% UBR race/ethnicity members.

The current VAT consists of 28 community members, ranging in ages from 18 to 76. Members reside across the US in locations ranging from suburban to rural in the states of Arkansas, California, New York, North Carolina, Louisiana, Ohio, Pennsylvania, Rhode Island, South Carolina, Tennessee, Texas, West Virginia, and Washington D.C. Several racial and ethnic groups are represented including Black/African American, Hispanic, White, American Indian, and Asian, and there is also representation for the sexual and gender minority (SGM) community (Fig. 1). VAT members represent groups with at least 9 chronic conditions and 23 affiliated advocacy organizations.

### Impact on the program

Since its inception, the *All of Us* Research Program has been influenced by the VAT in both tangible and intangible ways. In one example, the importance of a new participant survey was highlighted by a founding VAT member who is a person of color. The survey is focused on social determinants of health (SDOH) as one way to better understand the health of marginalized and underrepresented communities. SDOH are social, economic, and environmental factors that impact an individual’s overall health and well-being [36]. The additional survey enables researchers to correlate a participant’s health status with social and environmental factors. The VAT member’s input was part of the information relayed to program leadership to guide their decision on whether to add the survey. Although the VAT member’s input was not the sole reason the survey was added to the program, their input signifies how an engaged community can play an active role in public health research initiatives.

In another example, a VAT member, who is part of the SGM community, identified a scenario where study results that were accessible to the public could include a combination of data points that could potentially be used to identify *All of Us* participants who are members of the trans community. He voiced this privacy issue to the *All of Us* Governing Committee, which is responsible for reviewing and vetting research projects, and the board took the issue seriously. After several conversations about the potential breach of privacy, the Governing Committee recommended involving two members of the SGM community in the project to strengthen their voices and raise issues that may be otherwise overlooked. This action could set a precedent for how researchers engage minorities in research, highlighting the importance of including diverse communities in decision-making processes.

### VAT as an entry point

All *All of Us* participants are offered opportunities to volunteer for *All of Us*. However, VAT members are offered additional opportunities considering their unique role and experience within their community. For example, VAT members are invited to participate in the planning and development of program-wide messaging amplification initiatives for *All of Us*. VAT members who volunteer to be part of these activities are provided with the necessary training and resources to be successful within and beyond their local community (Table 1).

**Table 1.** List and descriptions of volunteer opportunities that are made available to virtual advisory team (VAT) members

Opportunity	Description
*Presentations*	In the *All of Us* Research Program, community-specific presentations were developed to raise awareness and generate recruitment for the program. Virtual Advisory Team (VAT) members who represented these communities were invited to speak as program ambassadors. Those who chose to be part of the virtual presentations received additional coaching to hone their presentation skills. A total of 10 VAT members conducted a presentation. Of those, 5 members presented more than once, for a total of 34 presentations that included a VAT member as a speaker. Members of the VAT also had the opportunity to present at in-person events as panelists in San Diego, CA, and Washington, D.C. Approximately 15 VAT members have participated as panelists for Face-to-Face in-person, national consortium meetings.
*Webinars*	*All of Us* has partnerships with various organizations, some of which opt to produce *All of Us*-related online webinars. VAT members are invited as guests to represent their community during the webinar.
*Guest Editor*	By volunteering as Guest Editors, VAT members have participated in the planning and design of the *All of Us* participant newsletter titled, *My Medical Minutes.* In one example, a Spanish-speaking VAT member served as Guest Editor on an issue that featured curated content to represent the Hispanic community. Additionally, another VAT member served as a Scientific Reviewer for the Spanish version of the newsletter to ensure the content was appropriately written and styled for Spanish-speaking communities.
*Serve on the National Community of Participant Ambassadors Board*	VAT members who have been consistent in their contributions and participation are invited to serve on the National Community of Participant Ambassadors Board, a national advisory board. In the last 5 years, 6 VAT members have been nominated for participation on this board.
*Participant testimonials*	VAT members have contributed both video and written testimonials about their participation in research to share their experiences and encourage others to join.

## Discussion

Community participation in health research, whether done in-person or virtually, is increasingly recognized as a process that can improve how all research studies are prioritized, designed, carried out, and applied to real-world scenarios [22]. To enhance participation efforts, community engagement should be an ongoing and iterative process with clearly defined and communicated community partner roles and bidirectional trust and transparency [37]. Diversity across advisory board membership is critical to ensure broad perspectives are considered to meet the needs and experiences of all demographics.

CABs (VATs in our specific case) are a proven model to achieve meaningful community engagement for various types of health research. Due to the persistent underrepresentation of individuals of non-European descent in clinical trials, along with the negative impact of social determinants of health, health disparities, and variations in medication responsiveness affecting these communities, it is crucial to involve diverse groups in the research process from the outset as advisory board members and participants [38].

### Lessons Learned

CPBR guidelines recommend early formation of a CAB, ideally while the research question and geographic focus are being developed [39]. Early integration of a CAB into the research design process can help create an equal power balance and develop trust between researchers and community members from the outset of the project. Also, offering hybrid (both in-person and virtual) meeting formats will help accommodate a broad spectrum of member preferences and their available resources. It is important for investigators to maintain flexibility to adapt to the board’s needs throughout the study. Creating a recruitment strategy that prioritizes diversity lends itself to establishing an advisory board in which multiple perspectives can be shared with the research team to address concerns and contribute to essential aspects of the program. Ensuring diversity across the research team is also an important factor so that perspectives from diverse individuals are included to study health disparities and inequities that persist in our society [40]. Table 2 contains a summary of best practices from this work.

**Table 2.** List of best practices for recruiting and engaging diverse advisor board members

Best practice	Description
Offer an inclusive and safe space for members	The VAT started in 2019 with only 4 members which has grown to our current cohort of 28 members that has taken years to build. Building trust among our members to offer a space where they are heard and feel valued has been critical to keeping members on year over year. This has also contributed to their willingness to refer other members to the VAT.
Recruit through community organizations	Leverage networks and trusted community organizations to identify and recruit advisory board members. These organizations have direct access to the diverse populations you aim to engage and can provide valuable insights and recommendations. Scripps team members reached out to various community-based organizations around the United States, we worked with organizations such as Patients Like Me (a national patient advocacy group) and Pyxis Partners who formed a network of funded and non-funded *All of Us* Community Partner Organizations at a national scale to communicate the VAT application opportunity.
Select individuals with experience as peer or patient advocates in diverse communities	Prioritize individuals who have firsthand experience advocating for peers or patients within diverse communities. Their insights and lived experiences can provide authentic representation and ensure that the advisory board is truly reflective of the populations served. VAT members included this type of information in their application and were selected based on this experience.
Create opportunities for personal connection	Foster an environment where advisory board members can build personal connections with one another. This can be achieved through team-building activities, social events, and informal gatherings. Strong personal connections can enhance collaboration and engagement. Each VAT meeting begins with a wellness check in where members can share personal anecdotes.
Set ground rules for meetings	Establish clear and inclusive ground rules for advisory board meetings to ensure a respectful and productive environment. Ground rules should promote open communication, active listening, and equitable participation, helping all members feel valued and heard. We accept that members will have different points of view, validate each individual perspective, and respectfully end discussions of disagreement. Ground rules are outlined in our onboarding materials and reviewed periodically.
Offer compensation	Recognize the time and expertise of advisory board members by providing appropriate compensation. This practice demonstrates respect for their contributions and encourages sustained participation. If financial resources are low, offer return of value to participants that fall outside of monetary compensation. For example, each VAT member’s bio is hosted on the Scripps Digital Trials Center website. VAT members were compensated a nominal payment each year; members who attended at least 33% of meetings and who made themselves available asynchronously received payment.
Share results how feedback/input was used	Maintain transparency by regularly sharing how the feedback and input from advisory board members have been utilized. This practice builds trust, validates their contributions, and shows that their efforts are making a tangible impact on the organization’s decisions and actions. VAT meeting notes are shared through a consortium-wide platform for *All of Us* Research Program leadership to review.
Connect members with leadership	Allow for members to interact with members of leadership when possible. For example, ask Principal Investigators to present to advisory board members and gather feedback. One of our VAT members participated in a panel discussion with Dr Eric Topol on personalized medicine which was streamed online.

### Future Steps and Considerations

While the overarching purpose of the VAT was to incorporate diverse community voices into research design and implementation, ensuring representation remains a significant challenge. The digital divide continues to limit individuals’ access to a variety of digital technologies, particularly those in rural, low-income, and otherwise marginalized communities. If someone doesn’t have access to broadband Internet to participate in a video conference, they can participate by phone and mechanisms to provide feedback asynchronously (e.g., via email, text, or other communication platform) should be considered. This approach could also help include individuals who want to participate in a CAB or VAT but don’t have time between work and family commitments to attend a scheduled meeting.

Additionally, investigators should be willing to share their experiences from their own outreach strategies to build a collective knowledge base, particularly for decentralized studies that rely heavily on digital technologies. Including VAT members to review the results of the research is another consideration, for that would further strengthen and amplify the investigator-participant partnership and ensure the information is presented in a clear manner for all represented communities.

## Conclusion

Input from UBR individuals and communities is critical in healthcare and medical research. The formation of a CAB (in our specific case, a VAT) is an effective method to bring communities from urban and rural regions and from diverse backgrounds together to inform culturally appropriate engagement strategies to promote diversity, equity, and inclusion in public health research. However, proper oversight and structure are key in keeping board members connected and engaged to effectively carry out their responsibilities. Furthermore, adapting to the needs of the CAB is an essential element for long-term success. The combination of CBPR and digital technologies has the potential to promote public health for all.
